# Differences in classification of COPD patients into risk groups A-D: a cross-sectional study

**DOI:** 10.1186/1756-0500-7-562

**Published:** 2014-08-23

**Authors:** Stefanie Zogg, Selina Dürr, David Miedinger, Esther Helen Steveling, Sabrina Maier, Jörg Daniel Leuppi

**Affiliations:** Medical University Clinic, Cantonal Hospital Baselland, Rheinstrasse 26, Liestal, CH - 4410 Switzerland; Medical Faculty, University of Basel, Basel, Switzerland; Institute of Human Movement Sciences and Sport, ETH, Zurich, Switzerland

**Keywords:** Chronic obstructive pulmonary disease, COPD assessment test, COPD risk groups, COPD severity grades, Exacerbations, Modified Medical Research Council dyspnea scale, New GOLD 2011 classification

## Abstract

**Background:**

The Global Initiative for Chronic Obstructive Lung Disease proposed in 2011 a new system to classify chronic obstructive pulmonary disease (COPD) patients into risk groups A-D, which considers symptoms and future exacerbation risk to grade disease severity. The aim of this study was to investigate the agreement between COPD risk group classifications using COPD assessment test (CAT) or modified Medical Research Council (mMRC) and severity grades or past-year exacerbations. Furthermore, physical activity across risk groups was examined.

**Methods:**

87 patients with stable COPD were classified into risk groups A-D. CAT and mMRC were completed. Severity grades I-IV were determined using spirometry and the number of past-year exacerbations was recorded. To test the interrater agreement, Cohen’s Kappa was calculated. Daily physical activity was measured by the SenseWear Mini armband.

**Results:**

Using CAT, 65.5% of patients were in high-symptom groups (B and D). With mMRC, only 37.9% were in B and D. Using severity grades, 20.7% of patients were in high-exacerbation risk groups (C and D). With past-year exacerbations, 9.2% were in C and D. Interrater agreement between CAT and mMRC (κ = 0.21) and between severity grades and past-year exacerbations (κ = 0.31) was fair. Daily steps were reduced in risk groups B and C + D compared to A (p < 0.01), using either classification.

**Conclusions:**

When classifying COPD patients into risk groups A-D, the use of CAT or mMRC and severity grades or past-year exacerbations does not provide equal results. Daily steps decreased with increasing COPD risk groups.

## Background

**Figure 1:**
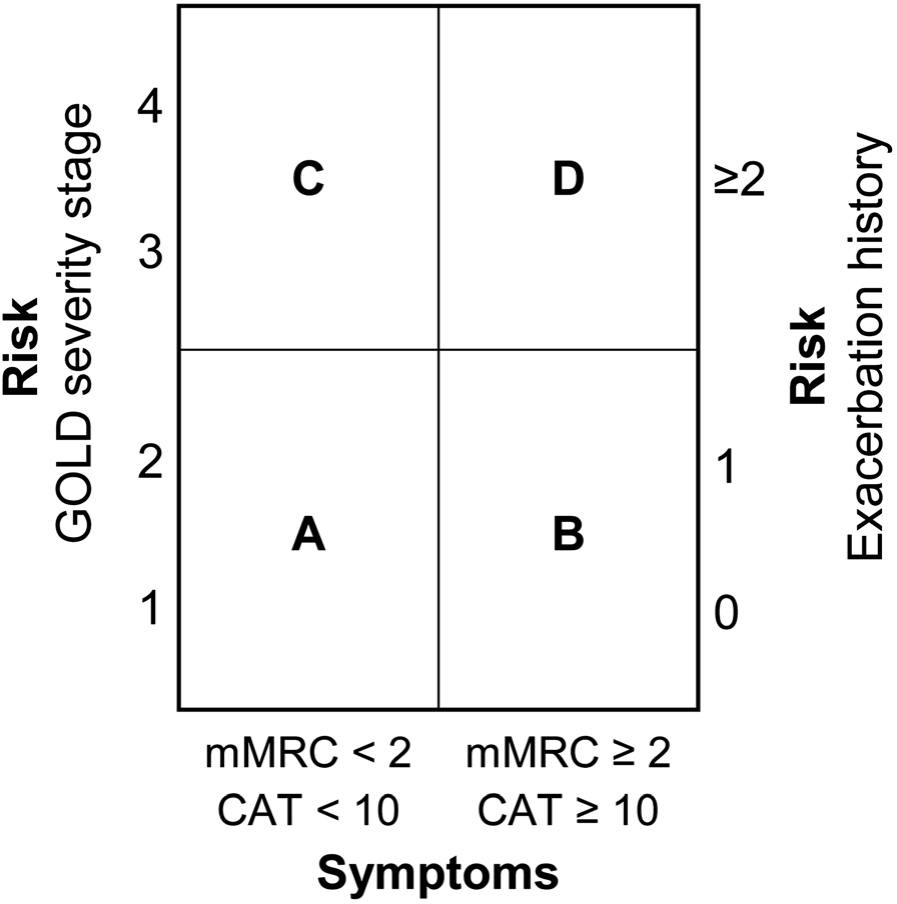
**Schematic illustration of the COPD classification into risk groups A-D.** CAT, COPD assessment test; COPD, chronic obstructive pulmonary disease; GOLD, Global Initiative for Chronic Obstructive Lung Disease; mMRC, modified Medical Research Council. Patient group A: Low risk, less symptoms. Patient group B: Low risk, more symptoms. Patient group C: High risk, less symptoms. Patient group D: High risk, more symptoms [1].

Chronic obstructive pulmonary disease (COPD) is one of the leading causes of mortality in most countries [1]. Based on the Swiss COPD cohort, 23-25% of patients with COPD experienced exacerbations requiring pharmacological treatment within one year [2–5]. While conventional COPD classification was mainly based on airflow limitation, the Global Initiative for Chronic Obstructive Lung Disease (GOLD) now recommends considering symptoms and exacerbation risk to grade disease severity into risk groups A-D (Figure 1). Symptoms are assessed by COPD assessment test (CAT) or modified Medical Research Council (mMRC) dyspnea scale. CAT ≥10 and mMRC ≥2 indicate high impact of symptoms (risk groups B and D). Exacerbation risk is determined by the degree of airflow limitation using spirometry-based severity grades I-IV or by the number of exacerbations in the previous 12 months. Patients in severity grades III-IV and those with ≥2 past-year exacerbations have a high exacerbation risk (risk groups C and D) [1].

Until now, there is little evidence concerning the agreement of these components determining the new COPD risk groups. Previous studies [6, 7] have shown that the threshold of CAT score ≥10 might not be equivalent to the threshold of mMRC score ≥2 for categorizing patients into low or high symptom groups. However, no study has investigated the agreement between risk groups classified either by severity grades I-IV or the number of exacerbations in the previous 12 months to determine future exacerbation risk. Lange et al. [8] have shown that regarding mortality, the overall trend was that low lung function was a stronger predictor of death than exacerbation history.

In patients with COPD, physical activity is related to pulmonary limitations [9–11], extrapulmonary effects [12], health-related quality of life [13] and individual lifestyle [14]. Furthermore, regular physical activity was found to reduce the risk of hospitalisations and exacerbations, and to modify smoking-related lung function decline [15, 16]. Moreover, physical activity level (PAL), daily steps and 6-min walk distance (6MWD) decline with increasing severity grades I-IV [9]. However, differences in physical activity across the new COPD risk groups A-D have not been investigated so far.

The primary aim of this study was to analyse the interrater agreement between COPD risk group classifications using CAT or mMRC, as well as using severity grades I-IV or past-year exacerbations. We hypothesized that all classifications yield similar risk group assignments. The secondary objective was to examine daily physical activity across COPD risk groups based on CAT or mMRC, and severity grades I-IV or past-year exacerbations. Furthermore, correlations between physical activity parameters and CAT score, mMRC score, number of past-year exacerbations and forced expiratory volume in 1 s in % of predicted (FEV_1_%predicted) were investigated.

## Methods

### Study subjects

**Figure 2:**
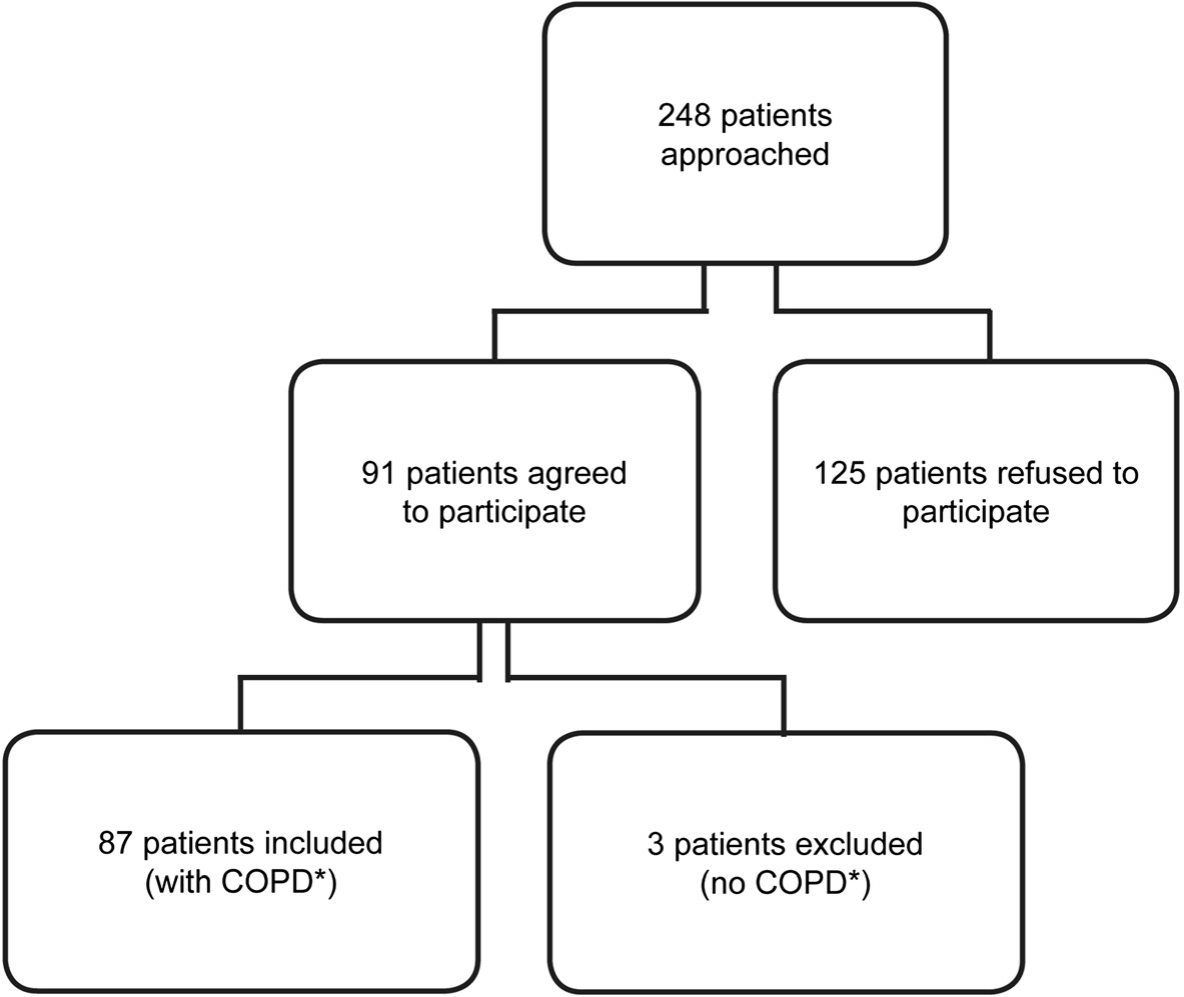
**Flowchart of study subjects.** *COPD was defined as post-bronchodilator FEV_1_/FVC ratio <0.70. COPD, chronic obstructive pulmonary disease.

From July 2011 to January 2012, patients with COPD were recruited from a patient-file database of the University Hospital Basel, Switzerland. Exclusion criteria were COPD exacerbations within the last 30 days and pregnancy. Ninety-one out of 248 approached patients agreed to participate (Figure 2). Reasons for refusal were: no interest, bad general condition, current hospitalisation and insufficient knowledge of the German language. Eighty-seven clinically stable COPD patients were finally investigated and classified into risk groups A-D according to the revised COPD GOLD guidelines 2011. The present investigation was approved by the local ethics committee (EKBB, 163/11) and written informed consent was obtained from all subjects.

### Study design

In this cross-sectional study, the Swiss German version of CAT and the mMRC dyspnea scale were administered to the patients in order to assess symptoms and exertional dyspnea, respectively. Then, spirometry was performed for determining COPD severity grades. In addition, exacerbation history and several demographic factors were recorded. Finally, patients were instructed to wear the SenseWear Mini armband for the subsequent week in order to quantify daily physical activity.

### Methods

#### COPD assessment test

CAT is a short health status questionnaire developed to provide a simple tool for assessing the impact of COPD [17]. It consists of 8 items, each presented as semantic 6-point differential scale, providing a total score ranging from 0–40 [17]. The content of CAT covers daily symptoms, such as cough, phlegm and chest tightness as well as other manifestations of COPD like breathlessness going up hills/stairs, activity limitation at home, confidence in leaving home, sleep and energy [17].

#### Modified Medical Research Council dyspnea scale

The mMRC dyspnea scale is a modified version of the original MRC dyspnea scale developed by Fletcher in 1952 [18]. It has more simplified statements and is based on 5 stages of exertional dyspnea ranging from 0–4 [19].

#### Lung function

Spirometry was performed according to the guidelines of the American Thoracic and European Respiratory Societies [20]. The EasyOne spirometer (ndd Medizintechnik AG, Zürich, Switzerland) was used to assess lung function before and 15 min after inhalation of 200 μg fenoterol. Severity of airflow limitation was classified according to the GOLD guidelines based on post-bronchodilator FEV_1_%predicted [1].

#### Exacerbation history

The number of COPD exacerbations in the previous 12 months was determined by asking the patients and consulting the hospital medical file. An exacerbation was defined as a worsening of the subject’s condition beyond normal day-to-day variations that required additional treatment with oral or intravenous corticosteroids or antibiotics [1].

#### Patients’ characteristics

Age, gender, height, weight, handedness, smoking status (yes, no, never) and current medication were recorded. Furthermore, the number of comorbidities was documented by accessing the hospital medical file.

#### Daily physical activity

Daily physical activity was measured by the SenseWear Mini armband developed by Bodymedia (Pittsburgh, Pennsylvania, USA) [21]. It integrates motion data from a three-axis accelerometer along with several other physiological sensors such as heat flux, skin temperature and galvanic skin response [21]. In patients with COPD, validity and reliability of the SenseWear armband were established by Hill et al. [22].

Participants were instructed to wear the SenseWear Mini armband on the left arm (triceps) for 7 consecutive days, except during water-based activities. The patients were told that the off-body duration of the armband should not exceed 1 h a day. To ensure a standardized procedure, the first and the last incomplete measurement day, including the study visits, were not taken into account. Therefore, the investigated measurement period was 5 days (3 weekdays and 2 weekend days). Reliability of this assessment period has been previously shown [23]. Patients with a wearing time of less than 5 days and less than 12 hours per day (from wake-up time to 12 hours after waking) were excluded from analyses [24, 25].

The physiological data collected by the armband’s sensors were processed by specific algorithms available in the software (professional software V.7.0, algorithm V.2.2.4). Age, gender, height, weight, handedness and smoking status were also considered in these calculations. Patients’ average daily number of steps, active energy expenditure (AEE), physical activity duration above 3 METs (PA_3_) and physical activity level (PAL) were examined. 1 MET defined as metabolic equivalent and expressing the energy cost of physical activity, corresponds to 3.5 ml/min/kg VO_2_ [20].

#### Missing data

In 2 patients, severity grades were determined with pre-bronchodilator data. In these 2 patients, asthma was excluded by asking the patients, if they had been previously diagnosed with asthma, and by looking at prior diagnoses of asthma in the medical files. Furthermore, the first 8 patients did not complete the mMRC dyspnea scale and in 9 patients, SenseWear data were missing for 1 or more days. They were therefore excluded from the corresponding analyses.

#### Statistical analysis

The main outcome measures were analysed using the SPSS software package (version 19.0, IBM, Germany). Significance was set at the 5% level. The Shapiro-Wilk test was used to test, whether data were normally distributed. Patients’ characteristics are presented as mean ± standard deviation (SD) or number and percentage. To test the interrater agreement between CAT and mMRC as well as between severity grades and past-year exacerbations, Cohen’s Kappa (κ) was calculated. Κ < 0.00 indicates “poor”, 0.00 ≤ κ ≤ 0.02 “slight”, 0.21 ≤ κ ≤ 0.40 “fair”, 0.41 ≤ κ ≤ 0.60 “moderate”, 0.61 ≤ κ ≤ 0.80 “substantial” and 0.81 ≤ κ ≤ 1.00 “perfect” agreement [26]. To analyse differences in demographic characteristics and physical activity across COPD risk groups based on CAT or mMRC and severity grades or past-year exacerbations, mean comparisons were performed using One-Way ANOVA, Kruskal-Wallis test or Chi-squared test, if appropriate. Risk groups C and D, both indicating high exacerbation risk, were combined for statistical analysis due to the small number of patients in these subgroups. Furthermore, Pearson correlations were calculated for parametric data and Spearman correlations for non-parametric data.

## Results

### Patients’ characteristics

**Figure 3:**
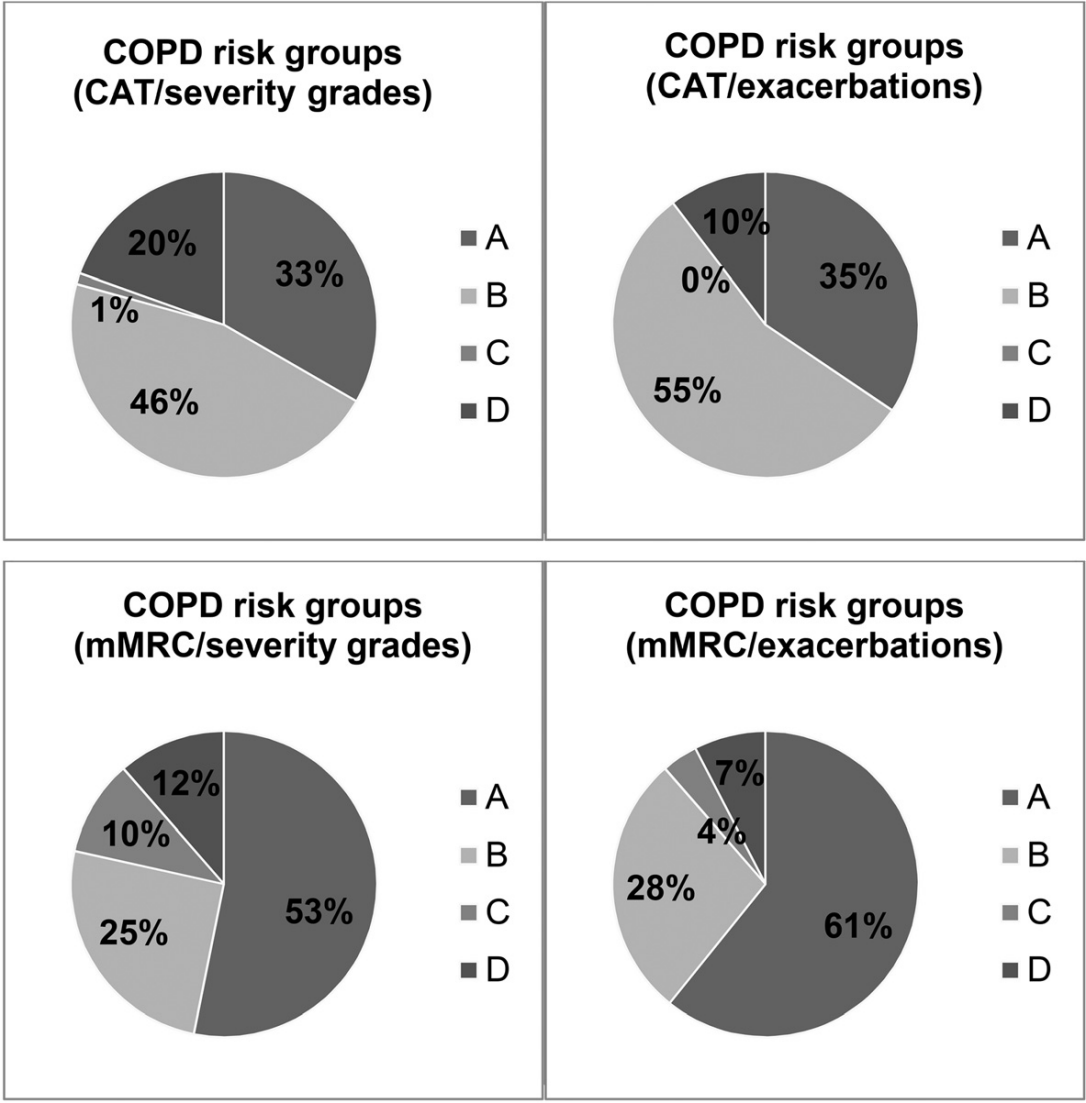
**Distribution of the 87 patients across the 4 possibilities of COPD risk group classifications.** CAT, COPD assessment test; mMRC, modified Medical Research Council.

Patients’ characteristics are presented in Table 1. Age ranged from 44–90 yrs (mean age: 67.3 ± 9.6 yrs). 51 men and 36 women were investigated. Almost half of the patients were current smokers, while 40 (46.0%) patients had stopped smoking and only 6 (6.9%) patients had never smoked in their life. The distribution of patients classified into risk groups A-D differed according to the use of CAT or mMRC and severity grades or past-year exacerbations (Figure 3). With CAT, 57 (65.5%) patients were found to be in high-symptom groups (B and D), compared to 33 (37.9%) patients using mMRC. Using severity grades I-IV, 18 (20.7%) patients were in high-exacerbation risk groups (C and D). With past-year exacerbations, 8 (9.2%) patients were in C and D.

**Table 1:** **Characteristics of the 87 study participants**

Variable	n (%) or mean ± SD
Age [yrs]	67.3 ± 9.6
Male	51 (58.6)
Current smokers	41 (47.1)
BMI [kg/m^2^]	25.5 ± 5.4
CAT score	13.3 ± 7.2
mMRC score^1^	1.4 ± 0.9
Number of past-year exacerbations	0.4 ± 0.8
FEV_1_predicted [%]^2^	69.1 ± 24.3
FEV_1_/FVC [%]^2^	53.3 ± 13.8
Number of comorbidities	2.2 ± 2.1
Average daily steps^3^	4783.6 ± 3337.6
Average daily AEE [cal]^3^	443.6 ± 383.1
Average daily PA_3_ [min]^3^	94.6 ± 84.0
Average daily PAL [METs]^3^	1.3 ± 0.3

BMI, body mass index; CAT, COPD assessment test; FEV_1_predicted, forced expiratory volume in 1 s of predicted; FVC, forced vital capacity; MET, metabolic equivalent; mMRC, modified Medical Research Council; PAL, physical activity level; PA_3_, physical activity duration above 3 METs; SD, standard deviation. ^1^(n = 79); ^2^(n = 85); ^3^(n = 78).

### Interrater agreement

The interrater agreement between CAT and mMRC was found to be fair (κ = 0.21). However, CAT and mMRC showed a significant correlation (r = 0.45, p < 0.001). Regarding severity grades and past-year exacerbations, a fair interrater agreement (κ = 0.31) was detected, too. FEV_1_%predicted and the number of past-year exacerbations correlated with each other (r = −0.29, p = 0.004).

### Demographic characteristics and physical activity across COPD risk groups

Demographic characteristics split by COPD risk groups based on CAT or mMRC and severity grades or past-year exacerbations are shown in Table 2. When comparing gender, smoking status and comorbidities across COPD risk groups, no significant differences were found. Basing risk groups on CAT and past-year exacerbations, age was higher in risk group A compared to B (70.4 ± 8.4 yrs vs. 64.7 ± 10.0 yrs, p = 0.029). Regarding the traditional GOLD grades I-IV, significantly less men were found to be in grade I compared to IV (p = 0.027), whereas all other characteristics did not differ across severity grades.

**Table 2:** **Demographic characteristics across COPD risk groups A-D and GOLD grades I-IV**

Variable		Age [yrs]	Male	Number of comorbidities	Current smokers
Classification	N	Mean ± SD	N (%)	Mean ± SD	N (%)
CAT - Severity grades	**87**				
A	29	70.2 ± 8.5	15 (51.7)	2.1 ± 2.6	13 (44.8)
B	40	65.6 ± 10.6	21 (52.5)	2.5 ± 1.9	22 (55.0)
C + D	18	66.2 ± 8.4	15 (83.3)	1.9 ± 1.6	6 (33.3)
mMRC – Severity grades	**79**				
A	42	66.2 ± 9.4	21 (50.0)	2.1 ± 2.2	23 (54.8)
B	20	69.9 ± 12.0	11 (55.0)	2.6 ± 1.7	10 (50.0)
C + D	17	66.1 ± 8.6	14 (82.4)	1.8 ± 1.6	6 (35.3)
CAT - Exacerbations	**87**				
A	30	70.4 ± 8.4	16 (53.3)	2.1 ± 2.6	13 (43.3)
B	48	64.7 ± 10.0	30 (62.5)	2.4 ± 1.8	24 (50.0)
C + D	9	70.7 ± 7.9	5 (55.6)	2.0 ± 1.9	4 (44.4)
mMRC - Exacerbations	**79**				
A	48	65.8 ± 9.2	26 (54.2)	2.1 ± 2.1	20 (41.7)
B	22	68.5 ± 12.1	15 (68.2)	2.4 ± 1.6	9 (40.9)
C + D	9	70.7 ± 7.9	5 (55.6)	2.0 ± 1.9	4 (44.4)
GOLD grades	**87**				
I	23	67.5 ± 10.2	9 (39.1)	2.1 ± 2.5	11 (47.8)
II	46	67.6 ± 10.0	27 (58.7)	2.5 ± 2.0	24 (52.2)
III	12	66.2 ± 9.2	9 (75.0)	1.5 ± 1.4	6 (50.0)
IV	6	66.2 ± 7.1	6 (100.0)	2.7 ± 1.9	0 (0.0)

CAT, COPD assessment test; COPD, chronic obstructive pulmonary disease; GOLD, Global Initiative for Chronic Obstructive Lung Disease; mMRC, modified Medical Research Council; SD, standard deviation.

Mean differences in physical activity across COPD risk groups are presented in Table 3. Basing risk groups on CAT and severity grades, steps were higher in risk group A compared to B (p = 0.003) and C + D (p < 0.001), while AEE showed a significant difference between A and B (p = 0.007). Using mMRC and severity grades, only steps were found to be reduced in risk group B (p = 0.001) and C + D (p < 0.001) compared to A. Based on CAT and past-year exacerbations, steps were higher in risk group A compared to B (p = 0.001) and C + D (p = 0.002), whereas AEE was different from risk group A to B (p = 0.007). Using mMRC and past-year exacerbations, steps were also higher in risk group A compared to B (p = 0.001) and C + D (p = 0.002). Furthermore, only steps were found to be significantly higher in GOLD grade I (p = 0.001) and II (p = 0.004) compared to IV. PA_3_ and PAL showed no significant differences across all COPD classifications.

**Table 3:** **Means of steps, AEE, PA**_**3**_ **and PAL across COPD risk groups A-D and GOLD grades I-IV**

Variable		Steps	AEE [cal]	PA _3_ [min]	PAL [METs]
Classification	N	Mean ± SD	Mean ± SD	Mean ± SD	Mean ± SD
CAT - Severity grades	**78**				
A	27	6763.1 ± 4005.1	599.3 ± 477.0	116.3 ± 89.9	1.4 ± 0.3
B	36	4152.6 ± 2571.9	337.2 ± 297.3	76.5 ± 76.9	1.3 ± 0.3
C + D	15	2732.8 ± 1362.5	418.9 ± 295.4	99.1 ± 85.4	1.4 ± 0.3
mMRC – Severity grades	**71**				
A	40	5971.3 ± 3302.5	460.4 ± 399.1	98.2 ± 84.0	1.4 ± 0.3
B	17	3633.8 ± 2996.1	376.3 ± 343.9	79.9 ± 88.4	1.3 ± 0.3
C + D	14	2811.4 ± 1378.2	426.5 ± 305.0	100.9 ± 88.3	1.4 ± 0.3
CAT - Exacerbations	**78**				
A	27	6763.1 ± 4005.1	599.3 ± 477.0	116.3 ± 90.0	1.4 ± 0.3
B	42	3923.7 ± 2488.5	344.0 ± 288.8	77.7 ± 76.4	1.3 ± 0.3
C + D	9	2858.3 ± 1439.1	441.3 ± 334.7	108.4 ± 92.1	1.4 ± 0.3
mMRC - Exacerbations	**71**				
A	43	5794.4 ± 3247.1	469.2 ± 391.5	97.7 ± 84.8	1.4 ± 0.3
B	19	3426.5 ± 2937.7	349.3 ± 322.7	80.2 ± 84.9	1.3 ± 0.3
C + D	9	2858.3 ± 1439.1	441.3 ± 334.7	108.4 ± 92.1	1.4 ± 0.3
GOLD grades	**78**				
I	22	6191.8 ± 3985.2	496.9 ± 434.8	96.1 ± 97.1	1.3 ± 0.3
II	41	4778.3 ± 3128.5	424.1 ± 388.0	92.1 ± 78.0	1.3 ± 0.3
III	9	3358.1 ± 1436.3	462.1 ± 345.5	116.4 ± 103.2	1.4 ± 0.3
IV	6	1794.8 ± 371.2	354.0 ± 211.7	73.0 ± 44.9	1.3 ± 0.1

AEE, active energy expenditure; CAT, COPD assessment test; COPD, chronic obstructive pulmonary disease; GOLD, Global Initiative for Chronic Obstructive Lung Disease; MET, metabolic equivalent; mMRC, modified Medical Research Council; PAL, physical activity level; PA_3_, physical activity duration above 3 METs; SD, standard deviation.

### Bivariate correlations

Table 4 illustrates bivariate correlations between physical activity parameters and CAT, mMRC, FEV_1_%predicted and past-year exacerbations. CAT and mMRC showed a significant relationship with all investigated SenseWear activity parameters, whereas FEV_1_%predicted and the number of past-year exacerbations were significantly associated only with daily steps.

**Table 4:** **Bivariate correlations of steps, AEE, PA**_**3**_ **and PAL with CAT, mMRC, FEV**_**1**_ **%predicted and exacerbations (n = 78)**

Variable	CAT score		mMRC score ^1^		Exacerbations		FEV _1_%predicted ^2^	
	r	p	r	p	r	p	r	p
Steps	−0.37	**<0.001**	−0.51	**<0.001**	−0.23	**0.021**	0.42	**<0.001**
AEE [cal]	−0.24	**0.016**	−0.30	**0.006**	−0.05	0.332	0.13	0.136
PA_3_ [min]	−0.25	**0.014**	−0.27	**0.011**	0.02	0.430	0.05	0.337
PAL [METs]	−0.18	0.058	−0.21	**0.038**	0.02	0.441	0.02	0.449

AEE, active energy expenditure; CAT, COPD assessment test; FEV_1_%predicted, forced expiratory volume in 1 s in % of predicted; MET, metabolic equivalent; mMRC, modified Medical Research Council; p, probability level; PAL, physical activity level; PA_3_, physical activity duration above 3 METs; r, correlation coefficient. Significant p-values are highlighted in bold. ^1^(n = 71); ^2^(n = 77).

## Discussion

The main findings of this study were that CAT and mMRC, as well as severity grades I-IV and past-year exacerbations showed only a fair agreement when they were used to determine patients’ COPD risk groups according to the new COPD GOLD guidelines. Moreover, daily steps differed significantly across risk groups A-D, regardless of which parameters the risk groups were composed.

The new COPD GOLD guidelines recommend two alternatives for the assessment of symptoms and exacerbation risk. Originally, CAT and mMRC, as well as severity grades and past-year exacerbations were thought to provide equivalent risk group classifications [1]. However, Jones et al. [6] and Kim et al. [7] have shown that a CAT score ≥10 might not be equivalent to an mMRC score ≥2, when classifying patients into low or high symptom groups. To improve the interrater agreement, they suggested using a cut-point of mMRC ≥1 [6, 7]. The present investigation confirmed the fair agreement between CAT and mMRC. Using CAT, more patients were categorised into risk groups B and D with a high impact of symptoms than using mMRC. Since CAT and mMRC do not provide the same COPD risk group classifications, it may be favorable to restrict to one symptom assessment tool. While CAT assesses the general health status of COPD [17], mMRC was developed to measure dyspnea [18]. A CAT score ≥10 has been shown to have a significant impact on daily life in patients with COPD [27]. Furthermore, patients with a CAT score ≥10 are likely to be breathlessness on most days and get exhausted easily [27]. Due to its comprehensiveness, CAT may be preferred for classifying patients into COPD risk groups A-D.

Based on severity grades, twice as many patients had a high exacerbation risk compared to the use of past-year exacerbations, when classifying patients into COPD risk groups A-D. Despite the clear definition of an exacerbation [28], it is still difficult to ensure a correct recording of the exacerbation history. On the contrary, spirometry-based severity grades represent an objective and reliable measurement [1]. The direct comparison of these two risk assessments might be difficult and could explain the discrepancy between them. Due to a higher validity and reliability, spirometry-based severity grades may be preferred for COPD classification into risk groups A-D.

Daily steps better distinguished patients with COPD across the new risk groups than AEE, PAL and PA_3_. This finding is underlined by the fact that daily steps correlated with all components (CAT, mMRC, FEV_1_% predicted and past-year exacerbations) of the new COPD risk groups. Furthermore, daily steps might reflect patient’s mobility and lifestyle better than PA_3_ and PAL. Engström et al. [14] could show that patients with COPD differed from healthy controls in both, walking activities as well as mobility. Previous studies [10, 29] confirmed that daily steps diminished with increasing COPD severity grades. In contrast, Watz et al. [10] showed that also PA_3_ and PAL decreased with increasing COPD severity grades I-IV, but no significant difference was found between severity grades I and II. In the present study, only few patients were in risk groups C and D, whereas Watz et al. [10] had evenly distributed number of patients in all severity grades. These findings suggest that PA_3_ and PAL may not differ between mild COPD severity grades I and II and risk groups A and B.

### Clinical implications

The new COPD GOLD guidelines propose specific therapy according to risk group classifications. Misclassification due to the use of CAT ≥10 or mMRC ≥2 and severity grades III-IV or ≥2 past-year exacerbations could lead to inconsistent management and treatment of the affected COPD patients. Therefore, it might be advantageous to use only one tool to assess symptoms and exacerbation risk, respectively.

As daily steps showed the strongest association with the disease, pulmonary rehabilitation could use daily steps to assess functional status in patients with COPD.

### Strengths and limitations

In this study, components of the new combined COPD risk groups were assessed in a standardized way by reliable assessment tools. No remarkable differences in demographic characteristics across risk groups were detected using either classification.

However, the sample size and the number of patients with high exacerbation risk were limited. Therefore, the present study was not representative for COPD risk groups C and D and the comparison between exacerbation risk using severity grades or past-year exacerbations may be biased. This could have led to the weak agreement between these two parameters. Further research with a larger sample size is needed to clarify this issue. Furthermore, the SenseWear Mini armband was found to underestimate the number of daily steps at slow walking speeds in patients with COPD [30]. The present analysis still found a significant association between daily steps and disease severity, which needs to be confirmed by further studies. Another limitation was the cross-sectional study design, which does not allow assessment of disease changes over time.

## Conclusions

The present analyses showed that the use of CAT or mMRC and severity grades I-IV or past-year exacerbations did not provide the same results when classifying COPD patients into risk groups A-D. These findings suggest that the new GOLD 2011 classification into risk groups A-D may require modification. Daily steps were significantly reduced in severe COPD risk groups compared to mild ones, while AEE, PA_3_ and PAL showed no or just a weak association with disease severity. Further investigation with a larger sample size is required to confirm our results.
